# Can experienced physiotherapists identify which patients are likely to succeed with physical therapy treatment?

**DOI:** 10.1186/s40945-015-0003-z

**Published:** 2015-07-08

**Authors:** Chad E Cook, Thomas J Moore, Kenneth Learman, Christopher Showalter, Suzanne J Snodgrass

**Affiliations:** 1 Department of Orthopaedics, Duke University, 2200 Main Street, 27705 Durham, NC USA; 2 Department of Physical and Occupational Therapy, Duke University Medical Center, Clinic 1E, Trent Drive and Erwin Road, 27710 Durham, NC USA; 3 Department of Physical Therapy, One University Plaza, 44555 Youngstown, Ohio USA; 4 Maitland-Australian, Physiotherapy Seminars, PO Box 1244, 11935 Cutchogue, NY USA; 5 Department of Physiotherapy, University Drive, 2308 Callaghan, NSW Australia

**Keywords:** Prognosis, Prediction, Physiotherapist, Musculoskeletal, Spine

## Abstract

**Background:**

The purpose of the study was to determine if clinician predicted prognosis is associated with patient outcomes.

**Methods:**

The study was a secondary analysis of data that were collected in 8 physiotherapy outpatient clinics. Nine physiotherapists with post-graduate training in manual therapy (mean 20.3 years of experience) were asked at baseline to project the outcome of the patients evaluated. In total, 112 patients with low back (74 %) or neck (26 %) pain were treated pragmatically with interventions consisting of manual therapy, strengthening, and patient-specific education. Outcomes measures consisted of percent change in disability (Oswestry or Neck Disability Index), self-reported rate of recovery (0–100 %), and percent change in pain (numerical pain rating scale). Hierarchical logistic regression determined potential factors (clinician predicted prognosis score (1–10) at baseline, dichotomised as poor (1–6) and good (7–10); symptom duration categorised as acute, subacute or chronic; same previous injury (yes/no); baseline pain and disability scores; within-session improvement at initial visit (yes/no); and presence of ≥ one psychological factor) associated with meaningful changes in each of the three outcomes at discharge (disability and pain > 50 % improvement, rate of recovery ≥82.5 % improvement).

**Results:**

Clinician predicted prognosis (OR 4.15, 95%CI = 1.31, 13.19, *p* = 0.02) and duration of symptoms (OR subacute 0.24, 95%CI = 0.07, 0.89, *p* = 0.03; chronic 0.21, 95%CI = 0.05, 0.90, *p* = 0.04) were associated with rate of recovery, whereas only clinician predicted prognosis was associated with disability improvement (OR 4.28, 95 % CI 1.37, 13.37, *p* = 0.01). No variables were associated with pain improvement.

**Conclusions:**

Clinician predicted prognosis is potentially valuable for patients, as a good predicted prognosis is associated with improvements in disability and rate of recovery.

## Background

Early prognosis is important for making clinical treatment decisions. Physiotherapists usually use a combination of decision making methods, including but not limited to hypothetico-deductive reasoning, intuition, and pattern recognition, when assessing prognosis and treatment [1, 2]. Pattern recognition is the categorization of patients based on exemplars from previous experience. It has been described as the non-analytic reasoning that contributes to clinical decision-making and it may increase clinician accuracy [3, 4]. Intuition or pattern recognition may assist physiotherapists in predicting patient prognosis early in the treatment process. This is supported by reports from physiotherapists that indicate they believe pattern recognition is an essential part of their decision-making [5, 6].

To our knowledge, the association between physiotherapist-predicted prognosis and various forms of patient outcomes has formally investigated in three studies. Hancock and associates [7] retrospectively examined the ability of physiotherapists to determine who was likely to have a quick resolution of pain in a population of patients with acute low back pain (*N* = 240). Prognosis assessment was performed at baseline and prior to treatment, and based on an 11 point scale (0 = very slow resolution of pain, 10 = very fast resolution of pain). The authors found that physiotherapists were able to identify those who would have quicker resolution, although not with the same accuracy as a clinical prediction rule. Recently, Abbott and Kingan [8] prospectively investigated the prognosis of a physiotherapists’ summative opinion on the long term functional outcome of individuals with a new episode of chronic or recurrent low back pain (*N* = 138). Physiotherapists formulated their opinion directly after performing a standardized physical examination and scored their prognoses on a 4 point Likert scale (1 = very good, very likely; 2 = good, moderately likely; 3 = poor, fairly likely; and 4 = very poor, very likely). Their investigation supported that physiotherapists were able to identify patients who had higher self-reported disability at 12 months.

Lastly, Dagfinrud and colleagues [9] examined the predictive ability of manual physiotherapists’ prognostic assessment in identifying patients with neck and low back pain (*N* = 157) at risk for having persistent disability (high score on Oswestry Disability or Neck Disability Indices). The authors [9] also compared physiotherapists’ findings to a standardized instrument (Orebro Musculoskeletal Pain Questionnaire, OMPQ). Although both the physiotherapists’ prognosis and the OMPQ were significantly associated with 8-week outcome on the Oswestry Disability Index (ODI) for low back pain patients, associations were weak and neither predicted outcomes for the neck pain patients. The study differed from that of Hancock *et al.*, [7] and Abbott and Kingan [8], in that the authors did not allow physiotherapists to experience a typical clinician-patient encounter (physiotherapists in the study were blinded to the results of the patient questionnaires), and they included patients with neck pain whereas the other two studies did not.

The aforementioned studies [7–9] appear to support a physiotherapists’ ability to identify the prognosis of patients with low back pain, specifically with respect to length of time for recovery for pain and disability scores. Mixed findings exist with respect to a physiotherapists’ ability to predict prognosis better than a standardized instrument or clinical prediction rule. Only Dagnifrud *et al.*, [9] examined prognosis in a mixture of chronicity levels, and none of the previous studies investigated the concept of rate of recovery, which is a patient derived self-assessment that is neither solely affiliated with pain or disability. Subsequently, the primary purpose of the current study was to determine whether clinician prediction of patient prognosis was associated with changes in disability, changes in pain and self-reported rate of recovery, when all assessment information is available to the physiotherapist at the initial consultation. A secondary aim was to explore the relationships between a physiotherapists’ predicted prognosis and other prognostic variables previously shown to be associated with patient outcomes.

## Methods

### Study design

This study was a secondary database analysis of a prospective cohort study [10] in which data were collected from May of 2011 to April of 2014. The prospective cohort study involved assessment of the concept of a “comparable sign”, and was observational. Because the original design was observational and required no prospective assignment of human participants or groups of humans to one or more health-related interventions to evaluate the effects on health outcomes, clinical trials registration was not required. All patients enrolled in the study signed an informed consent statement that was approved by the Walsh University Human Ethics committee in North Canton, Ohio.

### Eligibility criteria

#### Patients

All data were gathered in one of eight outpatient physical therapy clinics in the United States. For eligibility to participate in the primary study, patients were required to be 18 years of age or older with mechanically producible cervical or lumbar spine pain which occurred during clinical examination movements. All subjects also had to have required care beyond a single visit and had to speak English. Clinicians were instructed to target consecutive patients with spinal pain for inclusion into the study.

Exclusion involved the presence of any red flag (tumor, metabolic disease, rheumatoid arthritis, osteoporosis, prolonged history of steroid use) or signs consistent with nerve root compression that resulted in a radiculopathy (*i.e.*, diminished muscle stretch reflex, or diminished or absent sensation to pinprick in any upper or lower extremity dermatome). Additional exclusion criteria included a history of neck or low back related surgery or current pregnancy.

#### Physiotherapists

The study included 9 orthopedically-oriented physiotherapists, all of whom had rigorous, extensive training in manual therapy principles, orthopedic manual therapy certification, or were Fellows of the *American Academy of Orthopaedic Manual Physical Therapists*. The physiotherapists’ experiences ranged from 12 to 30 years (mean = 20.3 years) and practice settings were either hospital-based or private outpatient facilities. All were familiar with data collection in research projects and had experience collecting and recording data in two previous randomized controlled trials [11, 12].

### The examination and interventional process

#### Pre-study

Prior to involvement, all physiotherapists participated in a standardized, mandatory 30-minute educational webinar that explained the primary purpose of the study, the data collection methods, and the requirements for participation. Physiotherapists were also made aware of the secondary purpose of the study, which was to evaluate their ability to predict the projected outcome.

#### Initial visit

All physiotherapists performed a patient response-based examination [11] in which feedback was gathered with each targeted active or passive movement and subsequent treatment was a by-product of what was identified during the examination. A standardized examination process was used for all patients and the process involved analyzing movement patterns and pain during the examination phases of; 1) active physiological movements, 2) passive physiological movements and 3) passive accessory movements. All data captured during the initial visit was recorded immediately after completion of the encounter.

#### Week two and discharge

Throughout the bout of care, treatment interventions were performed pragmatically to ensure ecological validity and almost exclusively consisted of manual therapy, strengthening, and patient-specific education. Specific interventions were not the purpose of the study thus the components of each patient’s treatment were not collected. The physiotherapists collected outcomes data for disability and pain at week two and at discharge collected these along with the rate of recovery. Patients were discharged when the physical therapist felt they had meet their maximal improvement, when the patients self-discharged, or when the two parties mutually agreed on discharge. Discharge was not delayed for the sake of the study, thus in rare cases patient-encounters were shorter than the 2 week follow up.

#### Variables captured

At baseline, each physiotherapist recorded demographics (*e.g.*, age, race, gender, and diagnosis), duration of symptoms (categorized by acute < 6 weeks, sub-acute 6 weeks to 12 weeks, and chronic > 12 weeks), baseline outcomes measures for pain (Numeric pain rating scale) and disability (ODI or Neck Disability Index), previous history of a similar injury/sameness of symptoms(Yes or No), presence of a within-session change in pain or movement strategies (Yes for improvement, or No for no change or worsening symptoms), and presence of baseline psychosocial concerns (Yes or No).

All variables used in the modeling for this study were selected based on their previously investigated relationships with prognosis for either neck or back pain [13]. Age has been associated with poorer prognosis for subjects with neck pain [14, 15]. Longer duration of symptoms has been associated with a poorer recovery [16], whereas higher intensity of baseline levels of pain and disability has been associated with delayed recovery for patients with neck pain [17] and low back pain [15, 18]. A previous injury has been identified as the most prominent variable associated with recurrent low back pain [18], for first-time low back pain [19], and for poor outcomes with neck and back pain [15, 18, 20, 21] although to our knowledge the similarity (sameness) of the symptoms to the previous injury has not been formally investigated. A within-session change is an improvement in the patient’s pain or movement strategy that occurs during the initial visit [22]. A within-session change in either pain, movement, or both has been reported as a useful predictor of outcomes in previous studies [22–24].

Psychological factors have been associated with negative outcomes for subjects with neck [25] and back pain [26]. Presence of baseline psychosocial concern was based on any single positive answer from seven questions associated with enjoyment of employment, presence of a relationship with spouse or partner, depression, anxiety, social support systems, relationship with work colleagues, and use of medications for an unmentioned mental health condition. The tool used was novel and was created to provide a comprehensive assessment of psychosocial problems without overly burdening the patient with multiple psychological scales. The tool has not been analyzed for reliability or validity. Any positive finding from the seven was coded as “yes” whereas negative findings were coded as “no”.

At the initial clinical encounter, each physiotherapist was asked to estimate each patient’s potential for a successful outcome, based on their professional appraisal. Operationally, the physiotherapists were instructed to evaluate *all component parts* of their evaluation in their prediction of prognosis for the patient. Similar to the method used by Dagfinrud and colleagues [9], physiotherapists were instructed to score each patient on a continuum of 1 (suggesting a very poor projected outcome) to 10 (suggesting an excellent projected outcome) during the initial assessment. Each therapist was asked to score each patient following their complete encounter with the patient, including patient history, physical examination, treatment and reassessment. Upon examining the distribution of physiotherapists’ scores and using a receiver operating curve(ROC), the physiotherapist prediction of prognosis was dichotomised as a *good projected prognosis*(scores that range from 7 to 10), or a *poor projected prognosis* (scored as 1–6).

### Outcome measures

Primary disability measures included the ODI [27] or the Neck Disability Index (NDI) [28], whereas the primary pain measure was the numeric pain rating scale (NPRS). At discharge, the self-reported Rate of Recovery (RoR) was captured [11].

#### Oswestry disability index and the neck disability index

The ODI was used to measure patient disability in the patients with back pain. The ODI is a scale of 10 questions with scoring of 0–5 for each question, and the ODI defines disability as the higher the score, the greater the disability [27]. We used percentage change to determine the change score for each patient. This was calculated as [(baseline ODI score–final ODI score)/(baseline ODI score)] × 100 [29]. The NDI was used for the patients with neck pain, as it was designed for measuring pain related disability in this population [28]. The NDI contains ten focused sections. Seven items focus on activities of daily living. Each item is scored on a 6 point scale and can reach a maximum score of 5; therefore, the maximum score is 50 [28]. Content and construct validity and reliability of the NDI have been previously shown in patients with neck pain [30]. As with the ODI, we used percentage change to determine the change score for each patient. This was calculated as [(baseline NDI score–final NDI score)/(baseline NDI score)] × 100. Others have used a 50 % change from baseline as an appropriate discriminative threshold for disability scores in previous studies [29]. Thus, for analysis, the percent change in ODI/NDI was dichotomised as ≥ 50 % change (successful outcome) and <50 % change (not successful).

#### Numeric pain rating scale

The NPRS was used for patient perception of pain intensity using a scale of 0 (“no pain”) to 10 (“worst pain imaginable”). The NPRS has been found to be reliable and responsive [31]. We also used a percentage change as our outcome measure. This was calculated as: [(baseline NPRS score–final NPRS score)/(baseline NPRS score)] × 100. Greater than or equal to a 50 % improvement has been used by others [32] in different populations as an acceptable level of change indicating successful outcome. Thus for analysis, we categorized the percent change in NPRS as ≥ 50 % change (successful outcome) and <50 % change (not successful).

#### Rate of recovery

Self-reported rate of recovery was scored as (0–100 %) [11]. Patients responded to the physiotherapists asking them whether they were recovered and by how much by scoring their recovery on a scale from 0 % (meaning no recovery at all) to 100 % (meaning totally recovered). This scoring procedure is a variant of the single alphanumeric evaluation, and has been previously used with patients with shoulder pain [31, 33] and low back pain [11]. Previous work [34] has identified scores >82.5 % are related to global improvements in outcome. Thus, for analysis, we categorized the % recovery as ≥ 82.5 % improved (successful outcome) and < 82.5 % improvement (not successful).

### Number of observations per variable

Number of observations per variable was determined by using the recommendations of Homer and Lemeshow [35]. For simple univariate multinomial or logistic regression analyses, a minimum observation-to-variable ratio of 10 is recommended, although a number this low will likely overfit a model [35]. For this study, only eight variables were targeted.

### Data analysis

All analyses completed were performed using Statistical Package for the Social Sciences (SPSS), version 21.0 (IBM Corp, Armonk, NY). Intention to treat analysis was used, and for missing data at any follow-up time point, the last observation was carried forward. Descriptive statistics were used to describe the full patient sample. Frequencies of physiotherapists’ prediction of prognosis scores were evaluated for each physiotherapist to determine variations among practitioners. Linearity of effect of continuous variables was evaluated by plotting to identify potential curvilinear relationships. If curvilinear relationships were found, categories were created and were entered as ordinal data with a set of indicators (dummies). Individual estimates were then plotted to visualize linearity and checked if there are significant differences in the individual estimates.

To assess multicollinearity in the modeling and relationships among the 8 predictor variables, a correlation matrix was calculated for all independent variables. A correlational finding of *r* > 0.7 between independent variables was used to assess the potential of multicollinearity [36]. Analyses of continuous measures were performed with a Pearson Product Correlation. Analyses of dichotomous or categorical measures were performed with Cramer’s V whereas analyses of continuous to dichotomous or categorical variables were performed with a Biserial correlation. Cohen [37] characterized a correlation of 0.10 as depicting a small relationship, a correlation of 0.30 as a moderate relationship, and a correlation of 0.50 as a large/strong relationship. P values of <0.05 were considered significant.

Distinct hierarchical logistic regression analyses were performed for each of the dependent variables: percent improvement on patient-reported rate of recovery, ODI or NDI, and NPRS. Hierarchical models were used instead of stepwise modeling because automated stepwise models may sometimes lead to potential illogical conclusions and because the modeling used in this study was exploratory. For each analysis, individual *P* values, odds ratios and 95 % confidence intervals, and Nagelkerke values were reported. A Nagelkerke is a pseudo R square measure that investigates the usefulness of the model [38]. The value is similar in concept to the coefficient of determination (R^2^) in linear regression. The R^2^ statistics do not measure the goodness of fit of the model but indicate how useful the explanatory variables are in predicting the response variable and can be referred to as measures of effect size.

## Results

The study enrolled 83 (74.1 %) patients with low back pain and 29 (25.9 %) with neck pain. Of the 112, nearly all were Caucasian (95.5 %), a majority were female (*N* = 64; 57 %), and the mean age was 54.3 years (SD = 13.4 years). Slightly fewer than 50 % (48.6 %) reported a previous history of this same spinal condition. The baseline ODI was 32.8/100 (SD = 17.8) whereas the baseline NDI was 32.7/100 (SD = 16.8). The baseline pain scores were a mean of 5.76 (SD = 2.1) and individuals were seen for a mean of 10.4 total visits (SD = 8.3). The average duration of symptoms reported at baseline was 11.9 weeks (SD = 19.3). Fifty-two (46.8 %) of the individuals qualified as acute spinal pain, 31.5 % were sub-acute and 21.6 % were chronic. Slightly over 41 % (41.4 %) responded yes to one or more of the seven psychosocial questions. A total of 86.6 % of the individuals exhibited a within-session change during the examination. The mean raw physiotherapist prediction of prognosis value was 6.94 (SD = 2.02). For individual physiotherapists, mean raw prediction of prognosis values ranged from a low of 2.3 (SD = 0.57) to a high of 7.7 (SD = 1.15). Using the dichotomised variables discussed earlier (a score of 7–10), physiotherapists projected that 74.8 % of the patients would have a good prognosis.

Average ODI percentage change scores for patients with LBP were 64.6 % (SD = 35.9), average NDI percentage change scores for those with neck pain were 64.1 % (SD = 37.8), and average NPRS percentage change scores were 71.2 % (SD = 28.8) for those with LBP and 63.4 % (SD = 36.6) for those with neck pain. Rate of recovery percentages were 83.5 % (SD = 19.6) for LBP and 79.7 % (SD = 23.9) for neck pain.

When evaluating linearity of effect, only baseline pain demonstrated a non-normally distributed composition. However, when categorized, associations did not improve. Because there is a lack of an appropriate threshold within the literature for the categorization of the baseline measures used in the current study, the continuous variables collected at baseline were entered into the models without transformation.

For the correlation matrix (Table 1), small to moderate significant correlations were present among a number of variables. Physiotherapists’ prediction of prognosis was statistically significantly associated with pain at baseline, disability at baseline, age, and the presence of a within session change (all with small to moderate relationships). There were no instances in which multicollinearity was present and variable modification was not required prior to regression modeling.

**Table 1:** Correlation Matrix evaluating Association of predictor variables: nominal measures were calculated using Cramer’s V, continuous to nominal measures were calculated using a Biserial correlation, whereas continuous measures were analyses using Pearson Product correlations

*Variables*	Clinician prediction of prognosis	Duration	Previous injury	Pain baseline	Oswestry/Neck disability baseline	Age	Within session change	Psychological factor
Clinician prediction of prognosis								
Duration	0.41 *P* = 0.51							
Previous injury	0.28 *P* = 0.36	0.31 *P* = 0.95						
Pain baseline	0.20 *P* = 0.03	−0.09 *P* = 0.30	−0.20 *P* = 0.84					
Oswestry/Neck disability baseline	0.27 *P* < 0.01	−0.03 *P* = 0.75	−0.46 *P* = 0.64	0.46 *P* < 0.01				
Age	0.19 *P* = 0.03	−0.20 *P* = 0.84	−0.05 *P* = 0.61	−0.04 *P* = 0.69	0.17 *P* = 0.08			
Within session change	0.48 *P* < 0.01	0.47 *P* = 0.18	0.01 *P* = 0.94	0.28 *P* < 0.01	0.29 *P* < 0.01	−0.06 *P* = 0.55		
Psychological factor	0.18 *P* = 0.87	0.41 *P* = 0.49	0.05 *P* = 0.59	0.10 *P* = 0.29	0.09 *P* = 0.34	0.02 *P* = 0.85	0.01 *P* = 0.91	

Table 2 outlines the results for the logistic regression analysis with an outcome variable of rate of recovery. Physiotherapist prediction of a good prognosis and duration of symptoms were both associated with rate of recovery. Patients identified by the physiotherapists as predicted to have a good prognosis were 4.15 (95 % CI = 1.3, 13.19) times more likely to report a rate of recovery of greater than or equal to 82.5 % at discharge than those who were identified to have a poor prognosis. Further, compared to patients with acute pain, those with sub-acute and chronic conditions were less likely to report a rate of recovery score greater than 82.5 %.

**Table 2:** Final logistic regression model for factors associated with Rate of Recovery (patient report of ≥82.5 % improvement at discharge; *N* = 112, Nagelkerke = 0.32)

Predictor variable	Odds ratio (95 % confidence interval)	*P* value
Clinical prediction of prognosis	4.15 (1.31, 13.19)	**0.02**
Duration of symptoms		
Subacute	0.24 (0.07, 0.89)	**0.03**
Chronic	0.21 (0.05, 0.90)	**0.04**
Same previous injury	0.68 (0.24, 1.93)	0.47
Pain scale at baseline	0.98 (0.72, 1.31)	0.89
Oswestry/Neck disability index at baseline	1.01 (0.97, 1.04)	0.68
Age (years)	1.02 (0.98, 1.08)	0.29
A within session change during the initial visit	2.03 (0.42. 9.79)	0.38
Presence of at least one psychological factor	1.67 (0.59, 4.73)	0.33

Bold indicates statistical significance

The physiotherapists’ prediction of prognosis was also the only significant variable associated with a 50 % change in the disability status (ODI or NDI) with use of the logistic regression analysis. Patients identified by the physiotherapists to have a good prognosis were 4.28 (95 % CI = 1.4, 13.37) times more likely to report a 50 % improvement in disability at discharge than those who were identified to have a poor prognosis (Table 3).

**Table 3:** Final logistic regression model for factors associated with disability (≥50 % change on the Oswestry Disability Index or Neck Disability Index, *N* = 112, Nagelkerke = 0.18)

Predictor variable	Odds ratio (95 % confidence interval)	*P* value
Clinical prediction of prognosis	4.28 (1.37, 13.37)	**0.01**
Duration of symptoms		
Subacute	0.60 (0.18, 2.67)	0.60
Chronic	0.88 (0.28, 4.38)	0.88
Same previous injury	1.42 (0.51, 4.01)	0.51
Pain scale at baseline	1.20 (0.88, 1.63)	0.25
Oswestry/Neck disability index at baseline	0.98 (0.95, 1.02)	0.36
Age (years)	1.01 (0.97, 1.05)	0.58
A within session change during the initial visit	1.61 (0.39, 6.68)	0.51
Presence of at least one psychological factor	1.08 (0.38, 3.07)	0.88

Bold indicates statistical significance

There were no variables that were significantly associated with a 50 % reduction of pain (Table 4). Physiotherapist prognostic prediction provided an odds ratio of 3.96 (95 % CI of 0.99, 15.75) but the odds ratio crossed 1.0 and was not statistically significant.

**Table 4:** Final logistic regression model for factors associated with pain outcome (≥50 % improvement on the numerical pain rating scale, *N* = 112, Nagelkerke = 0.26)

Predictor variable	Odds ratio (95 % confidence interval)	*P* value
Clinical prediction of prognosis	3.96 (0.99, 15.76)	0.05
Duration of symptoms		
Subacute	0.38 (0.78, 1.81)	0.22
Chronic	0.75 (0.17, 3.40)	0.71
Same previous injury	2.61 (0.76, 9.01)	0.13
Pain scale at baseline	0.74 (0.53, 1.05)	0.09
Oswestry/Neck disability index at baseline	1.03 (0.98, 1.07)	0.22
Age (years)	0.96 (0.92, 1.01)	0.11
A within session change during the initial visit	1.82 (0.35, 9.39)	0.47
Presence of at least one psychological factor	0.70 (0.20, 2.44)	0.58

## Discussion

Our purpose for the present study was to determine whether the physiotherapists’ prediction of prognosis during the initial clinical encounter for the projected outcome of patients they treated was associated with the actual clinical outcome. In addition, we examined the relationship of physiotherapists’ prediction of prognosis to other prognostic variables that have been associated with clinical outcomes in previous literature, and modeled the overall relationship of these variables with the physiotherapists’ predicted prognosis. The findings suggest that experienced physiotherapists can project a recovery and disability outcome in patients with back and neck pain. This lends support to the value of a clinical judgment during prognostic assessment. In our study, physiotherapists’ prediction of prognosis was the only significant predictor variable associated with disability and one of two variables associated with rate of recovery. Although our primary finding is similar to that of others, our results are unique in several notable ways.

We selected a number of prognostic variables that were not included in the previous three studies [7–9], such as within session changes, sameness of previous injury, and a novel psychological instrument. However, none of these variables were significant in any of the models. Past work has suggested that a between session change is a much stronger predictor of outcomes than a within session change and lends support to the concerns that an immediate effect is truly a valuable finding within an examination [24]. Within session findings were related to the physiotherapists’ prediction of prognosis and it is likely that these findings are used to guide the physiotherapists’ the prognostic assessment but further study is needed. The concept of sameness of a previous episode is different than prior episodes (or number of prior episodes) in that we required patients to report a familiarity with their current bout of spine pain. Our novel psychological measure was an untested instrument and it may have failed to appropriately capture the most important constructs associated with delayed prognosis. It is worth noting that the previously investigated studies did find significance with measures such as the modified somatic pain questionnaire, catastrophizing, and fears avoidance beliefs, all of which were represented by specific questionnaire items within the novel tool.

The current study found that physiotherapists’ prognosis was a stronger predictor of clinical outcome (disability and rate of recovery) that the other prognostic variables used in our models. In contrast, Hancock *et al.*, [7] found that a clinical prediction rule of a) baseline pain, b) duration of current episode, and c) number of previous episodes was a stronger predictor than clinician judgment. One reason for the difference may be because we opted not to include number of previous episodes. Further, because this was a secondary analysis, the current study omitted several of the prognostic variables that have been investigated by others [7–9]. These include: work-related requirements, depression indices, and pain drawings [8]; spinal mobility measures [8, 9]; influence of catastrophizing, number of previous episodes, coping skills, or pain distal to the knee [7]; and the Örebro Musculoskeletal Pain Questionnaire [9]. Similar to previous studies, we did include duration of symptoms and it was the only variable other than the physiotherapists’ prediction of prognosis that was associated with outcome in any of the models; significantly associated only with the rate of recovery. Conversely, neither Dagnifrud *et al.*, [9] nor Abbott and Kingan [8] found significant associates with duration of symptoms and their outcomes.

The effect size of the physiotherapists’ prognosis in our study was larger than those previously reported. This may be reflective of 1) the differences in thresholds we used to determine success in our trial, 2) the skill set of our physiotherapists, or 3) the fact that we did not include a number of variables previously included in other studies. For the disability measures (ODI and NDI) we required a 50 % change from baseline measures to qualify as a success; a value that requires a notable overall improvement from baseline. Dagnifrud *et al.*, [9] required only a change of 10 points or greater in ODI and NDI scores (*i.e.*, 20 % improvement) from the raw baseline score. It is difficult to assess the comparability of our findings with Abbott and Kingan [8] and Hancock *et al.*, [7] because their selections for thresholds for disability and pain are notably different than those used in our study. Abbott and Kingan [8] retained the linearity of the Roland Morris Disability Index for their computational modeling. Hancock and associates [7] used number of days until a successful outcome (defined as a 1–10 pain score of 0 or 1 for seven consecutive days). The physiotherapists in our study were highly trained and were very experienced with a mean of 20.3 years of clinical practice and were similar in skills and experience to those of Abbott and Kingan [8] (17 years, all with post-graduate training in manual therapy). Hancock *et al.*, [7] and Dagnifrud *et al.* [9] did not report the skill level of their study clinicians. The additional training and years of experience of the physiotherapists in the current study may have contributed to their ability to make an accurate prognosis. However other factors, such as the variables selected for the models or the information available to the therapists at the time of prognosis may have been the key reasons for a larger effect size for physiotherapist prognosis.

Unlike Dagnifrud *et al.*, [9] but consistent with Abbott and Kingan [8] and Hancock *et al.*, [7] the physiotherapists in our study had complete information when making their prognostic assessment. Physiotherapists were told to take into account all aspects of the patient’s presentation, including demographics, patient history, the physical examination and results and their own experiences with similar patient presentations in the past. We were interested in the physiotherapists’ decision making ability when all these factors were present because this is similar to what occurs during a traditional patient-clinician encounter. Our findings suggest that outside of duration of symptoms, physiotherapist prognosis was a stronger predictor of outcome than the other variables that were investigated in our study. Further, variables that have demonstrated they are prognostic in previous studies were not significantly associated with our three different outcome constructs. We were somewhat surprised by these findings, especially since past works [14–24] have reported rather robust relationships. Nonetheless, by constructing a prognosis, potentially through use of comprehensive clinical information collected, the physiotherapists were able to determine the probable outcomes.

One other area of difference was that pain and disability scores at baseline were not related to the outcomes in the modeling we performed, which is a finding that is different from Hancock *et al.*, [7] and Dagnifrud *et al.* [9] (in Model B of their modeling scheme). Abbott and Kingan [8] did not formally investigate these measures. This difference may again be related to how we identified success for our outcome measures. By using percentage change from baseline, we marginalized the influence of the baseline raw score (severity), a confounding issue recognized previously [39].

### Limitations

There are number of limitations in this study. First, the same physiotherapists who made the prognostic estimates were also the individuals who provided care. There is a risk that physiotherapists could have subconsciously influenced the outcomes (resulting in a poorer or better outcome) based on the initial assessment of each patient. The current study incorporated prognostic findings that were represented in past literature; however, it is possible that other factors not represented in the models influenced outcomes. Another limitation is the experience and skill sets of each physiotherapist. All physiotherapists had similar, structured training and it is possible that the prognosis prediction was actually a by-product of a structured decision making model used by clinicians from a similar background or training. Although the tool we used for measuring prognosis was the same as the tool used by Dagfinrud *et al.* [9] we were slightly less restrictive on what qualified as a good prognosis (7–10 scores were considered to be associated with a good prognosis). Further, the generalisability of the findings from this study is questionable since the majority of physiotherapists have not had the same level of postgraduate training of those in this study.

## Conclusions

Physiotherapist prediction of prognosis is based on the theory that the physiotherapist can use previous knowledge obtained from multiple sources and experiences to come to an appropriate decision about the likely outcome of care. This study suggests that experienced physiotherapists’ prediction may be useful for determining prognosis.
